# Strangulated Hernia (Abstract)*Stenographically Reported for this Journal by C. C. Mapes, Louisville, Ky.At a recent meeting of the Louisville Clinical Society, Dr. W. O. Roberts read a paper on Strangulated Hernia (International Journal of Surgery, Vol. x., No. 6) which is epitomized for publication with the discussion, the latter not having appeared in print.

**Published:** 1897-12

**Authors:** W. O. Roberts

**Affiliations:** Professor of Surgery and Clinical Surgery in the University of Louisville, etc., etc., Louisville, Kentucky


### STRANGULATED HERNIA* (ABSTRACT).

By W. O. ROBERTS, M. D.

Professor of Surgery and Clinical Surgery in the University of Louisville, etc., etc., Louisville, Kentucky.

A short time ago I saw a patient sixty-five years of age, a thin, sparely built man, a merchant by occupation who, two days before, while making some repairs about his store, was suddenly seized with pain in the region of the umbilicus. He walked from his store, a short distance, to his residence and soon afterward became nauseated and vomited. He was subject to chronic constipation, and, as he had not had an evacuation for twenty-four hours, concluded that the cause of his trouble was constipation, and immediately took two compound cathartic pills. The pain continued and the vomiting increased, the pain being referred always to the umbilicus. That evening he sent for his physician, who could discover no evidence of a hernia, and thought it was a case of intestinal obstruction from fecal impaction. There was no elevation of temperature, but the pulse was increased in frequency, and the patient suffered considerable pain. The doctor gave him ten grains calomel at once, and in a short time two enemas; the first came away considerably stained with fecal matter, the second was not discolored. The next morning he was given another ten grains of calomel. During the day he continued to vomit, and about noon he is said to have vomited a considerable quantity of blood.

I saw him at eight o'clock in the evening of the second day of his illness. His abdomen was enormously swollen; he was bathed in a cold sweat; expression anxious; temperature 101 F.; pulse 130; voice husky. Passing my hand over his abdomen, I detected a tumor about the size of an almond in the right iliac fossa. I asked if he had ever had a rupture, or any pain referred to the region of this tumor, and was answered in the negative. There was no tenderness about the tumor. The doctor said he thought it was an enlarged gland. In manipulating the tumor, to my great surprise, it suddenly disappeared, and with the disappearance of the tumor pain subsided and within fifteen or twenty minutes the man was asleep. Two hours afterwards he had a copious action of the bowels, and three others during the night. The next morning the abdominal distension had almost entirely disappeared. The pulse was much improved, and I considered the man in a fair way to recovery. No untoward symptoms developed and in a few days he was up.

*Stenographically Reported for this Journal by C. C. Mapes, Louisville, Ky.

At a recent meeting of the Louisville Clinical Society, Dr. W. O. Roberts read a paper on Strangulated Hernia (International Journal of Surgery, Vol. x., No. 6) which is epitomized for publication with the discussion, the latter not having appeared in print.

This was a case of strangulated hernia with some unusual conditions, and one that I have not before encountered or heard mentioned, viz.: the vomiting of blood. Another unusual symptom was the absence of pain referable to the tumor. While pain is usually referred to the tumor in the beginning, and later to the umbilical region, there are some cases where there is no pain referred to the tumor at any time.

In the majority of cases of strangulated hernia the diagnosis is easily made. Following a strain or muscular effort, there is a sudden appearance of a tumor which is irreducible, tense, dull on percussion, tender, painful and without impulse on coughing. There will be the general symptoms of intestinal obstruction, viz.: nausea and vomiting, the vomited matter consisting at first of the contents of the stomach, then of the duodenum, finally stercoraceous material. Constipation is usually complete from the moment strangulation occurs, so that not even flatus passes; occasionally, however, there is an evacuation from the portion of the bowel below the constriction. The patient has severe pain referred usually to the tumor first, then to the umbilical region; the abdomen is tympanitic; pulse frequent and feeble; countenance anxious; surface clammy; finally hiccough occurs with cessation of pain; rapid sinking of their vital powers and death.

Occasionally the foregoing symptoms are so modified that a most careful examination is necessary to establish the diagnosis. Strangulation may occur at the internal inguinal ring and the tumor be so small as to render its detection impossible. Such cases usually give a pre-existing history of hernia. If there is much omentum in the sac, tension will not be marked; if there is a double hernia on the same side, the posterior one may be strangulated and tension masked by the lax state of the anterior one. While vomiting is a prominent symptom of strangulation, it may be absent, or occur only two or three times, and then only when the patient moves or takes food or liquids into the stomach. "Extensive peritonitis with copious effusion of a puriform liquid may occur without any pain, and with but little tenderness and no elevation of temperature, the anxiety of countenance and sharpness of pulse being the only symptoms that lead to its existence," and death may occur from exhaustion consequent upon vomiting without any sign of gangrene in the constricted portion of the intestine.

In incarcerated hernia there is constipation and vomiting, but not to the extent which occurs in strangulation; the impulse on coughing is not absent; pain in the tumor and region of the umbilicus not so marked, and the strength of the patient is but little affected.

In inflamed hernia, while the tumor is tender and painful, the impulse is present and symptoms of obstruction do not exist. In cases of double hernia there may be some difficulty in determining which is the seat of strangulation, but usually the diagnosis can be made with certainty from the local symptoms. If necessary, operate on both hernias. Agnew records an instance in which a woman with three irreducible hernias, two inguinal and one femoral, was attacked with the usual symptoms of strangulation, it being impossible to discover which one was involved. Herniotomy was done on all three, the last one proving to be the one affected. "If the patient be a female, and there be present an inguinal and femoral rupture, offering alike negative signs of strangulation, the femoral should be selected for operation as being the most likely to cause the trouble." (Agnew). In pregnancy and threatened miscarriage, vomiting, while persistent, is never stercoraceous; constipation is not complete, and if there is a tumor, the impulse on coughing will be present.

Treatment: The indications are to relieve the constriction and return the contents of the hernia to the abdominal cavity, provided gangrene has not occurred and the vitality of the part is not destroyed or so crippled that recovery is impossible. Reduction should be accomplished as soon after strangulation occurs as possible. Delay is exceedingly dangerous, and to wait until fecal vomiting is culpable. "Opium will sometimes mask or mitigate all the symptoms of strangulation, and physicians are not infrequently led to fatal delays by the implied security it gives, the surgeon not being called until after irreparable injury has been inflicted upon the tissues and death is already imminent." While in the passive form of strangulation it may be several days before gangrene of the constricted portion takes place, it will occur in a few hours in the active variety. The danger is greater when the tumor is small than when it is large, and in femoral than in inguinal hernias. There are two methods of accomplishing reduction, viz.: taxis and herniotomy. Taxis is only to be practiced in the earliest history of the case. It is not to be attempted if there be signs of local change, such as inflammation, emphysema or bruising about the hernial protrusion, or if symptoms have been very acute, *i. e.*, such as great pain, excessive vomiting and signs of collapse, or if the distinct symptoms have continued for two or three days, or when strangulution occurs in old irreducible hernias. Taxis should be done with great gentleness, as otherwise irreparable injury may be inflicted upon the tissues.

"I may represent the danger which is associated with violent attempts to reduce the hernia, by stating that more irreparable damage may be inflicted on the bowel in a few minutes by coarse, impetuous, brute force, than natural means of constriction could produce in several days" (Buckett). The dangers attending taxis, outside of bruising the tissues, are rupture of the gut, rupture of the sac, and escape of the bowel into the sub-serous connective tissue, the protruding portion still remaining strangulated, and reduction of the hernial tumor *en masse* without relief of the strangulation. When under taxis the tumor disappears with sudden slip and a gurgle, the surgeon may be satisfied the hernia is reduced properly. Always practice taxis under an anesthetic, and with the patient on the table. Complete anesthesia overcomes muscular contraction and straining of the patient, and lessens the danger of injury to the protruded portion. The foot of the table should be raised ten or fifteen inches, or an improvised Trendelenberg table made by putting a chair upside down on a kitchen table. By this position we secure gravitation of the abdominal viscera toward the diaphragm which is a valuable auxiliary to taxis. Manipulation of the tumor had best be made as follows: "Work the fingers of one hand around the neck of the tumor where it issues from the abdomen, holding its bulk in the palm of the hand if possible, and instead of trying to push the tumor back into the abdomen try to draw it further down. Now with the other hand grasp the canal with its contents (if inguinal hernia) gently but firmly between the thumb and fingers and while making traction and compression with the hand that is holding the tumor, manipulate the canal with a kneading motion. When you push upward on a strangulated hernia usually you carry it up over the edge of the ring upon the abdominal wall and accomplish nothing more. In the method suggested by traction you lengthen out the mass that is blockading the canal, favoring the effect which you afterwards produce by compression, that is the partial emptying of the engorged blood vessels, the displacement of imprisoned gases and fluids. This is further aided by the action of the fingers upon the canal which tends to work the bowel free at the point of constriction" (De Garmo).

Before attempting taxis, the patient should be advised that if you are unable to reduce the hernia, herniotomy will be performed at once. Under the present methods of operating the danger is very little greater, and the advantages are immense. The contents of the hernia can be inspected and the accidents attending taxis before mentioned are not possible. Adhesions and conditions likely to perpetuate the hernia can be dealt with, and means can be used to bring about a radical cure or at least to place it in a favorable condition.

DISCUSSION.

DR. J. M. KRIM: A word as regards treatment of strangulated hernia: I was called recently in an emergency case to see a man who had been able to reduce his hernia on previous occasions, but this time all efforts had failed, and strangulation had existed about eighteen hours before I saw him. I suggested that an operation should be done immediately as the man was vomiting, having cold, clammy sweats, etc., and he was taken to the infirmary at once and operated upon. The strangulated portion of the gut, which was about five inches in length, was very black and apparently dead. The operator thought there were two points of slough in the strangulated portion of the gut, yet he did not feel as if a resection should be carried out. He opened the gut and stitched it to the abdominal walls and left it. The question is whether a resection under such circumstances should not be done. The man died ten days later.

DR. LOUIS FRANK: I am sorry Dr. Roberts did not enter more fully into the surgical treatment of strangulated hernia, and lay more stress upon the surgical methods of treatment in preference to that by taxis. We should try to make differentiation between an incarcerated and a strangulated hernia. Further, we should, before we attempt to reduce a hernia by taxis (this being always done under an anesthetic), obtain the consent of the patient to be operated upon immediately by herniotomy if the taxis is unsuccessful. The results of taxis, which should always be limited as to the duration of time it is employed, will depend very largely upon the character and condition of the patient when the attempt is made.

As to the question of resection or stitching the suspected gut to the abdominal walls: We ought to be prepared at all times to do a resection if it is needed. It becomes a serious question in many cases just what we should do. If the resection is properly done, it adds little to the dangers of the operation. The resection may have to be done high up. If on account of gangrene we have stitched the gut to the abdominal wall, making a fistulous opening high up, it will be necessary to soon perform another operation or the patient will starve to death.

Dr. Robert's case emphasizes the fact that we should always make a very careful examination of patients where hernia is suspected or known to be present; further, it shows that a very slight amount of strangulation may give rise to very serious symptoms. In this case the hernia was so small that the patient had not noticed it, nor had it been discovered by the family physician.

DR. AUGUST SCHRACHNER: It is to a certain extent, a matter of guess-work, as to just where the hernia in Dr. Robert's case was located, whether it occurred at the internal ring or in one of the retro-peritoneal fossæ, some of which are in the region to which the essayist has referred the tumor. The location would very readily explain why there would be no tumor, or at least a very small one, perceptible, the hernia being in one of the fossæ of the peritoneum.

As to the vomiting of blood, that was likely due to a rupture of a gastric vessel. The man probably had little in his stomach and intestinal canal, which made the contractions very firm. The blood must have come from this source In analyzing the symptom of vomiting in hernia or in intestinal obstruction, you must take into consideration the portion of the alimentary canal which is involved, the further away from the mouth that the hernia occurs the later would be the vomiting; we must also consider the amount of contents which the bowel holds at the time of reception of the injury. Of course, the more the bowel contains the earlier the symptom of vomiting occurs.

In strangulated hernia it is difficult to say at the time just what is best for the patient, whether to do a resection or leave a loop of the intestine on the outside and make an opening, or to leave a loop of intestine on the outside and so place it that whatever may happen no harm will result to the interior; if the vitality is restored it may be dropped back into the abdominal cavity as we sometimes do in resecting the intestine. This procedure has been carried out a number of times, placing the gut on the outside of the abdomen, with suitable precautions in the way of moisture to keep the bowel in good condition, then later when vitality is evident the loop is dropped back into the cavity. The methods of intestinal resection have been so improved that it is now possible to resect the gut in many instances where formerly this was not deemed advisable. There are several methods of intestinal resection that can be done in a short space of time—the Woelfler or Maunsell's—so that resections to-day are common, and we resort to this treatment with less hesitancy than formerly.

DR. T. P. SATTERWHITE: In the connection with the case reported by Dr. Roberts, I desire to briefly relate two obscure cases which occured in the City Hospital some time since when I was surgeon to the female ward. A woman was sent from the medical ward for me to determine whether she had any trouble of a surgical nature that demanded relief. The left side embracing the whole iliac region was inflamed and somewhat œdematous and quite tender; there was no tumor demonstrable; firm pressure could not be made to determine this point, but by gentle manipulation no tumor could be made out; the surface had the appearance of an erysipelas of a mild character. In the course of a week or two the lower third of the left side of the abdomen sloughed away and fecal matter was discharged through the opening. During the entire time she was in the surgical ward she had diarrhœa. She died, and post-mortem showed a portion of the calibre of the bowel had been forced out through the internal ring which was constricted. Sloughing had occurred and the juices of the bowel had penetrated the cellular tissue causing the condition described.

Another case occurred in the City Hospital in which a woman was brought in with considerable pain, she said, at first, but which had subsided when admitted to the Hospital. Her pulse was exceedingly feeble, hardly perceptible; she was perfectly conscious, no vomiting and no pain at the time I saw her. There was in the inguinal region a little swelling about the size of a small hazelnut. I thought it was a hernia and called in consultation, some of the other members of the surgical staff. I remember Dr. Holloway responded and after an examination decided there was no hernia and proposed to defer operative interference until the next morning. The woman was then almost pulseless. She was put on the table and I cut down upon the tumor, which was found to be a lump of fat about the size of my thumb. Dr. Holloway and several others were present at the operation, and the doctor being very jubilant over his diagnosis remarked that he felt certain there was no hernia, etc. I transfixed the little mass and applied a ligature very tightly, expecting to remove the small tumor; fortunately I took my scalpel and opened it after having it transfixed, and there was the gut. I immediately united the ligature, and then completed the operation for hernia. The bowel was very black and showed evidence of beginning necrosis. The woman's condition was alarming; she took chloroform badly, and was gotten off the table as soon as possible. I did not believe she would live half an hour. The gut was returned to the abdominal cavity, and the woman recovered. The point I wish to make in reporting these two cases is that it would be interesting to know how black the gut may get before it is in a phacelous condition, and to what extent the bowel may become black and still retain its vitality, or regain its vitality after the constriction has been overcome. In the last case I have mentioned, the constriction was removed, and the patient recovered, although the bowel was perfectly black. No one expected her to live, and no time could be given for resection or stitching; we thought she was virtually a dead woman.

Dr. W. O. Roberts: The last case mentioned by Dr. Satterwhite is what is known as Littre's hernia, where only a segment of the bowel is constricted and its calibre is still open. Such cases are difficult to diagnose because of the absence of symptoms of constipation. Frequently in such hernia there is diarrhœa instead of constipation. Usually, however, there is local pain and other conditions that indicate the necessity for an operation. If operative interference had not been resorted to in the doctor's last case it would probably have resulted fatally.

You can not always tell this at once, and where there is any doubt as to whether the protruded portion is gangrenous I think it well to leave it out for a little while to see whether any change takes place in its color and appearance. It is always better to do a resection than to make an artificial anus, if the patient's condition will admit of it.

The case I have reported is one of the few cases of strangulated hernia that I have been able to reduce by taxis, where strangulation had existed for any length of time. I have frequently reduced strangulated hernias which were seen a few hours after strangulation occurred, but where they have been out for any length of time I have always failed by taxis.

